# Validation of the Human–Animal Interaction Scale (HAIS) in Czech Language

**DOI:** 10.3390/ijerph17207485

**Published:** 2020-10-15

**Authors:** Kristýna Machová, Veronika Juríčková, Tereza Nekovářová, Ivona Svobodová

**Affiliations:** 1 Department of Ethology and companion animal science, Faculty of Agrobiology, Food and Natural Resources, Czech University of Life Sciences, 16500 Prague, Czech Republic; nekovarova@af.czu.cz (T.N.); svobodovai@af.czu.cz (I.S.); 2 Department of Applied Neuroscience and Neuroimaging, National Institute of Mental Health, 25067 Klecany, Czech Republic; veronika.jurickova@nudz.cz; 3 First Faculty of Medicine, Charles University, 12108 Prague, Czech Republic; 4 Ethology and Ecology Research Group, Department of Zoology, Faculty of Natural Science, Charles University in Prague, 12800 Prague, Czech Republic

**Keywords:** human animal interaction, evaluation, scale

## Abstract

Human–Animal interaction (HAI) refers to any contact between humans and animals. Despite the lack of standardized measures of evaluation, one possible tool is the Human Animal Interaction Scale (HAIS). This study aimed to evaluate it in Czech language and to verify its use in clinical settings. One group of participants included 85 non-clinical volunteers; the second included 22 clinical participants, who were hospitalized in a long-term inpatient department All participants filled out the HAIS, the Companion Animal Bonding Scale (CABS) and the Companion Animal Semantic Differential (CASD). The Czech HAIS achieved similarly good psychometric properties as the original scale. The Cronbach’s alpha showed strong internal consistency (α = 0.920) in the sample of volunteers, but low internal consistency (α = 0.656) in the group of clinical participants. In non-clinical volunteers, all scales and subscales correlated mutually at the *p* < 0.01 level. In the group of clinical participants, the CABS did not show significant correlations with other scales and subscales, nor was there a correlation of total HAIS score with the perceived rapport with animals. The findings of this study suggest that the Czech HAIS may be an effective tool for evaluating HAI with non-clinical contingents, however careful modification is suggested before clinical use. One reason for this is the difficulty in conducting some activities assessed by the scale in a clinical practice or hospital setting.

## 1. Introduction

The term human–animal interaction (HAI) denotes any occasional or regular contact with an animal or pet ownership. One form of HAI could be an animal-assisted therapy (AAT) as a part of the therapeutic process in hospitals [1]. Animals are often involved in a clinical treatment or diagnostics and they seem to be helpful in the healing process [2]. AAT is a structured, goal-oriented therapy, which intentionally involves the involvement of animals in health, education and social programs to improve the effects of therapy, overall physical or mental health [3], the quality of clients’ life [4] or health-related problems [5].

Although AAT is not yet a conventional adjunctive therapy, its positive benefits are already seen in several inpatients, therapeutic, educational or socially oriented settings [6]. AAT is often incorporated into treatment and convalescence programs in various forms for clients with cognitive, emotional or social difficulties, or for people with physical disabilities [7]. AAT is a part of the therapeutic process in hospitals [8], hospices [9], rehabilitation, residential care facilities [10] and prisons [11,12]. AAT may also involve a wide range of patients—from patients with physical disabilities (e.g., cerebral palsy) to patients suffering from post-traumatic stress disorder [13], affective [14] or anxiety disorders [14,15], schizophrenia [14], dementia [16] or addiction to psychoactive substances [17].

Animals can also play a crucial role in children’s cognitive and social development [18], and they are also effective in the treatment of impairments in social interactions and functioning [5]. AAT may also reduce stress [18] and several symptoms of pervasive developmental disorders [5] in hospitalized children. The positive effect of AAT has been found in children with Attention-Deficit/Hyperactivity Disorder [19], autism [20] or Down syndrome [21]. 

Activities with animals are involved as a means of influencing balance and coordination [22], motor skills [23], communication skills [24], affective mood [14] or non-verbal communication [25]. AAT may also contribute to improving the patient’s attention, alertness, respiratory control and overall physical interaction [26], and horses have been found to increase self-control, motivation [26] and involvement in social activities [23]. In general, animals can enhance psychological as well as physiological well-being [5]. A meta-analysis of Nimer and Lundahl showed a moderate effect size in improving outcomes in medical difficulties, behavioral problems, emotional well-being and autism-spectrum symptoms [2]. Specific therapies differ in the goal of the therapy (physical, mental, emotional or social), choice of animal, form, structure, length, duration and number of administrations (they can be one-off or repeated). AAT can be practiced with various animals, but the dog is generally the most involved animal in therapy, mainly due to its social skills and easy training [17]. Other animal species involved in AAT are cats, birds, horses, dolphins, rabbits [22], elephants [21] or other small mammals and exotic animals [22,27,28,29]. However, according to international guidelines (e.g., IAHAIO), exotic animals such as dolphins, elephants, etc. should not be involved in animal-assisted interactions (AAI), which include AAT [30]. Each of these animals has some specific characteristics that can be employed to achieve desired therapeutic goals. 

The effects of AAT are usually evaluated by self-reported questionnaires (e.g., mood, stress level or self-esteem) or objective physiological measures (e.g., heart rate, blood pressure or cortisol level). The behavioral aspects of interaction between animal and human and vice versa are usually not measured [1]. Some authors evaluated the impact of AAT via interactions, such as playing, grooming, holding or talking with animals observed during therapy, which can have an important effect for both, human and animal [29,31]. Fournier et al. mentioned the absence of standardized measures to evaluate HAI as a potential reason for a lack of information in behavioral aspects of HAI and AAT [1]. 

To fill that gap, they constructed a tool to measure the behavior during HAI, of which AAT makes a part, called The Human–Animal Interaction Scale (HAIS). Items of HAIS were designed for generality, simplicity and flexibility of use [1] to reflect the range of behavior that may be observed during human–animal interaction. The scale evaluates the human-behavior (14 items) and the animal-behavior (10 items) subtotals, as well as overall total HAIS score in a desirable and undesirable way. The scale development and validation study of HAIS contained four groups with a total of 295 volunteers. The instrument showed a strong internal consistency overall (α = 0.82) and across subscales (αs > 0.72). Test–retest coefficients ranged from 0.36 to 0.94, with most coefficients at or above 0.70 [1]. To verify the convergent and divergent validity of the measure, The Companion Animal Bonding Scale (CABS) [32] and The Companion Animal Semantic Differential (CASD) [33] were used. Construct validity was provided by the observation of human–dog interactions by trained researchers. Validity was consistent across companion and small caged animals, and it seems to be an adequate behavior-based measure of HAI for the potential used for researchers, counselors and clinicians [1].

Measuring HAI, especially during AAT, is important for evaluating the possible change in the human–animal relationship, especially for assessing the impact of these interactions in psychological effects in the clients and also for assessing whether these interactions are pleasant for the animals. In the case of regular HAIS scaling within the AAT, it is possible to assess whether the animal is still happy with the interactions and is active or observe a decrease in friendly behavior. It is also possible to assess the relationship between the client and different species of animals or between individuals within one species. The aim of this study was to validate the psychometric properties of the Czech translation of The Human–Animal Interaction Scale (HAIS).

## 2. Materials and Methods

### 2.1. Participants and Procedure

The first sample included 85 non-clinical volunteers aged from 20 to 85 years (M_age_ = 38.39; SD_age_ = 21.06). This group of participants consisted of 71 (83.5%) women and 14 (16.5%) men; 70.6% of the group achieved secondary education and 29.4% higher education. Volunteers were recruited from the university campus of Czech university of Life science and rated the scales based on their last interaction with their own animal. Exclusion criteria were current hospitalization for any health problems and any psychiatric diagnosis. Volunteers were recruited using a snowball method of sampling.

The second sample included 22 clinical participants from The Military University Hospital in Prague to verify the use of the scale in clinical settings. Clinical participants were hospitalized in a long-term inpatient department without serious cognitive or psychiatric disorders. The group consisted of 16 (72.7%) females and 6 (27.3%) males aged from 48 to 96 years (M_age_ = 82.95; SD_age_ = 13.59). One respondent achieved basic education (4.5%), 14 (63.6%) respondents achieved secondary education and 7 (31.8%) respondents achieved higher education. Clinical participants underwent the canine-assisted therapy (CAT) during their hospitalization regularly with a handler and a therapeutic dog. The interaction was guided by the handler (but it was left to the client’s will how the interaction with the dog took place) and lasted 20 min for each clinical participant. CAT took place once a week and each participant in this group underwent 4 previous visits before compilation of scales. Mostly, it was about stroking the head and the whole body, serving treats or the presence of a dog on the bed with a client who touched it. After the CAT, clinical participants completed the scales and evaluated the interactions with the animal and their relationship to it. The participation in the research was voluntary, without any honorarium. All respondents signed an informed consent to participate in the study and to process the data for research purposes. The study was approved by the ethics committee of the Central Military Hospital in Prague and the Czech University of Life Sciences.

### 2.2. Measures

The Human–Animal Interaction Scale (HAIS) is a 24-item self-reported scale developed to assess human–animal interaction. The scale was designed to “describe and quantify behavior performed by human and nonhuman animals during an episode of interaction”. The scale was originally tested across various species, including companion animals (i.e., dogs and cats), caged animals (i.e., rats, rabbits and hedgehogs) and horses. Respondents rate the extent (5-point Likert scale, verbally coded as 0 = not at all, 2 = moderate amount and 4 = a great deal) of various human and animal behaviors, that may be perceived as desirable (e.g., petting and kissing an animal) and as undesirable (e.g., making a mess and aggressive behavior). The scale provides an overall total score, as well as animal-behavior (Animal_total_) and human-behavior (Human_total_) subtotal scores [1]. 

The Companion Animal Bonding Scale (CABS) is an 8-item behavioral scale to rate the extent (5-point Likert scale; 1 never, 5 always) of child–animal interactions. The scale was designed with a childhood focus and a contemporary focus. For contemporary bonding, all items were retained, with a three-factor solution (1, bonding or involvement factor; 2, factor related to animal size; 3, factor related to the companion animal’s responsiveness and autonomy) [32]. 

The Companion Animal Semantic Differential (CASD) [33] consists of 18 bipolar semantic differential word pairs, adapted from Osgood, Suci and Tannenbaum [34] with 7 new items. The measure was designed for accessing the respondent’s affective perception of a childhood companion animal. The scale has six divisions coded from 1 to 6, with 7 reversed items [33]. 

### 2.3. Study Design

To verify psychometric properties of the Czech version of HAIS, the same procedure and study design adopted in the original study was replicated to allow for an adequate comparison of results. The Czech versions of The Companion Animal Bonding Scale (CABS) [32] and The Companion Animal Semantic Differential (CASD) [33] were used to attest the validity of the HAIS. All scales used in our study were translated by a professional translator; back translation was done by the authors of this article and their colleagues. The participants filled out all scales in Czech language. Descriptive demographics questions and other additional questions about the experience and the ownership of animals were added. Data collection was realized in the Czech University of Life Sciences in Prague and in the Military University Hospital in Prague. The scales were administered to the respondents using the pencil–paper method. Volunteers were recruited by the snowball method of sampling (due to the primary goal of research aimed at verifying the properties of the Czech translation, without the ambition of generalization to a wider population, this method can be considered as sufficient). 

### 2.4. Statistical Analysis

Data were analyzed using statistical software IBM SPSS Statistics 23.0. The analysis of the results was focused on verifying the reliability and validity of the HAIS in two sample groups. Cronbach’s alpha coefficient was used to measure the internal consistency of all used scales. The Shapiro–Wilk test showed that the data significantly deviate from a normal distribution in both groups. For this reason, we used the nonparametric statistical analyses to calculate the differences and correlations of all the scales used in research groups. Differences between research groups (clinical vs non-clinical participants) were analyzed using the Mann–Whitney test. Spearman’s correlational analyses were used to prove the convergent and divergent validity of the scale, using the Czech versions of The Companion Animal Bonding Scale (CABS) [32] and The Companion Animal Semantic Differential (CASD) [33]. 

## 3. Results

In total, 107 respondents participated in the study—a group of non-clinical volunteers (n = 85) and a group of clinical participants (n = 22) who underwent the CAT during their hospitalization. The frequencies of responses to supplementary information and questions are presented in Table 1. The studied groups significantly differed in age (Mann–Whitney; U = 92.0, *p* < 0.001), but not in educational level (U = 927.5, *p* = 0.943). There were no significant differences between groups in the happiness of contact with animals (U = 753.5, *p* = 0.766), but clinical participants reported significantly more that they established rapport with animals (U = 613.0, *p* = 0.039). The group of clinical participants reported significantly less bad experiences with animals (U = 451.0, *p* < 0.001), less fear of animals (U = 427.0, *p* < 0.001) and less phobia of animals (U = 757.5, *p* = 0.047) than the group of volunteers. It is significantly more than in the second sample (U = 606.0, *p* = 0.020). 

Significant differences between groups were found only in the CASD score, but not in the other administered measures. The internal consistency of the used scales was measured with Cronbach’s alpha. All scales showed good internal consistency, except for total HAIS in the group of clinical participants (α= 0.656). The results are presented in Table 2. 

As in the previous study, the scale’s convergent validity was determined by the comparison of the HAIS and the CABS, and the CASD was used to verify the divergent validity of HAIS. In the group of non-clinical volunteers, all scales and subscales correlated mutually at the *p* < 0.01 level. The results of the correlational analysis are presented in Table 3. In the group of clinical participants, the CABS did not show significant correlations with all scales and subscales (see Table 4), nor was the correlation of total score of HAIS with the perceived rapport with animals significant (see Table 5). 

## 4. Discussions

The purpose of the study was to verify the reliability and the validity of the Czech translation of The Human–Animal Interaction Scale (HAIS) [1] using the Czech version of The Companion Animal Bonding Scale (CABS) [32] and The Companion Animal Semantic Differential (CASD) [33]. For better comparison of our results with the original study, the same scales, design and research procedures were used. The behavioral component of HAI in both animal and human was evaluated. The Czech version of HAIS showed satisfactory psychometric properties in non-clinical volunteers and it seems to be an effective tool for assessing human–animal interactions. In clinical participants, lower internal consistency was found.

Our study included two study groups. The first group contained 85 non-clinical volunteers, who evaluated the interaction with their own dog. The second sample contained 22 clinical participants hospitalized in a long-term inpatient department, who underwent a canine-assisted therapy. Respondents in the studied groups did not significantly differ in educational level. Clinical participants were significantly older than volunteers. The Czech version of the HAIS achieved similarly good psychometric properties as the original scale. The Cronbach’s alpha showed strong internal consistency (α = 0.920) in the sample of volunteers, but low internal consistency (α = 0.656) in the group of clinical participants. The possible reason is that some of the activities assessed by the scale are not allowed or hard to practice in a hospital (e.g., feeding or training the animals, taking pictures of animals). 

There are no scientific articles on this topic, but we have observed this issue during the practical use of HAIS in clinical settings. The next step, therefore, seems to be the appropriate adaptation of the HAIS scale to the given environment and animal species. In the case of the environment, the observed activities are different in wards with a special regime, or psychiatric wards, where it is not possible to have a phone and therefore take photos, etc. The client’s age and related ability to work with technologies can also be influencing, as well as the species involved and the objectives of the therapy. For example, in the case of horses, it is difficult to evaluate the item “makes friendly sounds”. Further, AAT with dogs can take place outside when it is possible for the client. These AATs focus on, e.g., throwing the ball and bending for it, overall activation and joy of the client. The goal of the therapy is to practice gross motor skills. Therapy and interactions with the dog are very often with good feedback from the clients, but HAIS would not have to reflect them sufficiently in its items because of lack of several different interactions. 

To verify the convergent and divergent validity of the scale, the scales CABS and CASD were used. In the original study, CABS was more strongly correlated with HAIS human-behavior items than the total score and did not correlate with animal-behavior items [1]. The CASD was designed to assess people’s attitudes concerning a pet [33]. Fournier et al. hypothesized that there is a difference in perception of one’s own pet and an unknown animal, so they expected the negative correlation between HAIS and CASD [1]. However, the factor of the presence of an own animal or an animal participating in the AAI is questionable. As mentioned in the literature, for example, walking dogs is very dependent on the motivation of the owner [35] and their relationship to the dog. In contrast, hospitalized patients can often feel lonely and the hospitalization can bring them boredom and stereotype, but the regular contact with a dog occurs to establish an emotional relationship. Surely, this topic should be better explored in the future [36]. 

In our study, total HAIS score positively correlated with all scales and the subscale correlated at the *p* < 0.01 level with acceptable coefficients (0.37–0.95) in the group of volunteers. In the sample of clinical participants, the total HAIS score significantly correlated with all used measures, except for CABS. Moreover, CABS also did not show significant correlations with other administered scales in the group of clinical participants. However, the small sample could also have influenced the results. In the case of CASD, non-significant correlation was found only with the animal-interaction subscale in the sample of clinical participants. In the group of volunteers, CASD significantly correlated with all scales and the subscale at *p* < 0.01 with acceptable coefficients (0.50–0.62). 

Even though the data provide the evidence of reliability and validity of the measure, data do not provide normative information and they cannot be generalized to a wider population. Our study included only two study samples, with a small number of participants. In addition, we did not evaluate HAI in participants involving more species of animals, only dogs. Some animals are inherently more interactive than others [1] and there is also no possibility to practice some activities with animals during sessions in hospitals, prisons or other AATs. For this reason, the results of various samples, species of animals or settings are needed to prove validity and reliability of the scale. Moreover, CABS and CASD have also not been validated in the Czech language yet so it also could be a limitation of the study. We presumed that this study is the first effort to validate all used scales in this field into the Czech language. Their validation is needed to verify the adequacy of their use in Czech language. AAT often takes place in several hospitals or day care centers in the Czech Republic, so the evaluation of HAI in AAT could be beneficial. 

Thanks to adequate psychometric properties of the scale, it can be used in various conditions—basic or applied research, and various samples and species of animals. HAIS may be helpful for evaluating the positive or negative change in the human–animal relationship and if these interactions are pleasant for the animals. It is also possible to assess the development of relationship between the client and different species of animals, or between individuals within one species, as mentioned in the Introduction. The measure can assess the human behavior toward animals to predict pet adoption or success of canine-assisted therapy. Moreover, HAIS can predict animals’ behavior toward humans also, and therefore can be used to evaluate the suitability and preparedness for their participation in therapy. Although the results were not significant in all cases, the HAIS tool may have several benefits for the usage. HAIS can be also used to evaluate the association between HAI and the mental and physical health of animals, as an animal-health diagnostic tool [1]. However, further analyses are needed. 

### Limitations

There could be a difference between relationships between own animals and animals which come for a visit. This relationship also involves interpersonal differences in the relationship between man and his own animal. In the case of clinical participants, this is the preselection of clients who have already agreed in advance with contact with the animal and can therefore be expected to be people with a minimally positive attitude towards the animal. Hospitalized patients, however, do not take the presence of the animal for granted and spend more time alone and are without a family. However, when animals come regularly, patients can develop a more lasting relationship with them. The difference between clinical participants (e.g., different diagnosis or different length of hospitalization) is another possible limitation of the study. In addition, the sample of respondents is small, and we cannot generalize the conclusions. Moreover, we did not apply power analysis to define the sample size. Participants were recruited by the snowball method of sampling, and the participation was voluntary. 

CABS and CASD have also not been validated in the Czech language yet, so that also could be a limitation of the study. We used these scales to follow the original HAIS validation procedure. These scales are intended for child respondents, but for our purposes we consider them suitable for their comprehensibility for older respondents and simple administration. Longer scales could have a negative effect on the concentration of older respondents when filling them out.

Another limitation could be the point of view of evaluation of the interaction with animals. It can be accessed as the frequency of activities or the willingness to perform them. Despite some physical barriers of clinical patients, their willingness and motivation can be significant.

To verify the properties of the Czech translation of all used scales, without the generalization of the results to wider population, we believe this study will be sufficient and useful. However, for statistical certainty, more psychometric research is needed in this area. 

## 5. Conclusions

It seems that HAIS in Czech language is an effective tool for assessing human–animal interactions. Its psychometric properties are, for now, suitable when used for evaluation of human–animal interaction in non-clinical participants. However, there are some difficulties with the assessment when used in a clinical setting mainly because some activities cannot be performed in social or healthcare facilities. Some questions would also be difficult to evaluate if animal species other than dogs were involved. In future studies, it will be important to focus on the evaluation of the questionnaire suitability after removing or changing some questions. It seems to be useful to adapt the HAIS scale to the environment where it will be used (e.g., for the case of outdoor, indoor or restricted activities), as well as variants taking into account the behavioral manifestations of the animal participating on AAT. For future utilization of HAIS, it seems that it would be appropriate to adapt the scale for use in clinical settings not just in the Czech, but also in the original language.

## Figures and Tables

**Table 1:** Supplementary information and questions.

Supplementary Information and Questions	Non-Clinical Volunteers (%)	Clinical Participants (%)
Dog owners	77.6	50.0
Cat owners	35.3	27.3
Other animal owners	37.6	18.2
More than one animal owners	43.5	9.1
Any animal owners	8.2	13.6
Want to owe an animal, but cannot	31.6	59.1
Having animal indoor	82.9	76.2
Having animal outdoor	47.6	42.9
Having animal indoor and outdoor	48.8	45
Bad experience with animals	48.1	0
Phobia of any animal	23.5	4.5
Fear of any animal	58.3	4.5
Allergy to any animal	11.8	0
Happy for the contact with animal during session (0–4 scale)	M ^1^ = 3.63 (SD ^2^ = 0.82)	M = 3.63 (SD = 0.68)
Establishing the rapport with animal during session (0–4 scale)	M = 3.66 (SD = 0.86)	M = 3.42 (SD = 0.77)

^1^ M, mean; ^2^ SD, standard deviation.

**Table 2:** Means, standard deviations, Cronbach’s alpha and between group differences.

Scale	Non-Clinical Volunteers		Clinical Participants		Mann–Whitney Test (*p*)
	M ^1^ (SD ^2^)	α	M (SD)	α	
HAIS	49.64 (14.51)	0.920	47.64 (14.52)	0.656	0.568
1.1 Human_total_	32.94 (9.97)		32.77 (9.58)		0.997
1.2 Animal_total_	16.69 (5.57)		14.86 (6.76)		0.243
CASD	93.39 (11.14)	0.886	85.32 (5.38)	0.800	<0.001 **
CABS	27.71 (7.81)	0.851	28.82 (7.15)	0.801	0.568

Human_total_, human-behavior subtotal score of HAIS; Animal_total_, animal-behavior subtotal score of HAIS; ^1^ M, mean; ^2^ SD, standard deviation; α, Cronbach’s alpha; ** *p* < 0.01.

**Table 3:** Correlations between scales in non-clinical volunteers (n = 85).

Scale	1	1.1	1.2	2	3	4
1.HAIS						
1.1 Human_total_	0.951 **					
1.2 Animal_total_	0.874 **	0.700 **				
2. CASD	0.621 **	0.608 **	0.534 **			
3. CABS	0.603 **	0.648 **	0.447 **	0.500 **		
4. Happy for contact	0.467 **	0.473 **	0.392 **	0.392 **	0.366 **	
5. Rapport	0.530 **	0.545 **	0.404 **	0.461 **	0.534 **	0.438 **

Human_total_, human-behavior subtotal score of HAIS; Animal_total_, animal-behavior subtotal score of HAIS; ** *p* < 0.01

**Table 4:** Correlations between scales in clinical participants (n = 22).

Scale	1	1.1	1.2	2	3	4
1. HAIS						
1.1 Human_total_	0.938 **					
1.2 Animal_total_	0.927 **	0.796 *				
2. CASD	0.519 *	0.650 **	0.417			
3. CABS	0.353	0.405	0.292	0.370		
4. Happy for contact	0.690 **	0.725 **	0.523 **	0.608 **	0.215	
5. Rapport	0.450	0.468 *	0.427	0.717 **	0.361	0.600 **

Human_total_, human-behavior subtotal score of HAIS; Animal_total_, animal-behavior subtotal score of HAIS; ** *p* < 0.01; * *p* < 0.05

**Table 5:** Scales and subscales correlations with HAIS total score.

Scale	Non-Clinical Volunteers		Clinical Participants	
	r_s_	*p*	r_s_	*p*
Human_total_	0.648 **	<0.001	0.938 **	<0.001
Animal_total_	0.447 **	<0.001	0.927 **	<0.001
**CASD**	0.500 **	<0.001	0.519 *	0.013
**CABS**	0.603 **	<0.001	0.353	0.108
Happy for contact	0.366 **	<0.001	0.690 **	0.001
Rapport	0.534 **	<0.001	0.450	0.053

Human_total_, human-behavior subtotal score of HAIS; Animal_total_, animal-behavior subtotal score of HAIS; r_s_, Spearman’s rho; ** *p* < 0.01; * *p* < 0.05.
